# Hanging by a thread: unusual nocturnal resting behaviour in a jumping spider

**DOI:** 10.1186/s12983-021-00410-3

**Published:** 2021-05-17

**Authors:** Daniela C. Rößler, Massimo De Agrò, Elia Biundo, Paul S. Shamble

**Affiliations:** 1 John Harvard Distinguished Science Fellows Program, Harvard University, Cambridge, MA 02138 USA; 2 Department of Biogeography, Trier University, Trier, 54295 Germany

**Keywords:** Anti‐predator adaptation, Suspended resting, Salticidae, Sensory ecology, Silk use

## Abstract

**Background:**

For diurnal animals that heavily rely on vision, a nocturnal resting strategy that offers protection when vision is compromised, is crucial. We found a population of a common European jumping spider (*Evarcha arcuata*) that rests at night by suspending themselves from a single silk thread attached overhead to the vegetation, a strategy categorically unlike typical retreat-based resting in this group.

**Results:**

In a comprehensive study, we collected the first quantitative field and qualitative observation data of this surprising behaviour and provide a detailed description. We tested aspects of site fidelity and disturbance response in the field to assess potential functions of suspended resting. Spiders of both sexes and all developmental stages engage in this nocturnal resting strategy. Interestingly, individual spiders are equally able to build typical silk retreats and thus actively choose between different strategies inviting questions about what factors underlie this behavioural choice.

**Conclusions:**

Our preliminary data hint at a potential sensory switch from visual sensing during the day to silk-borne vibration sensing at night when vision is compromised. The described behaviour potentially is an effective anti-predator strategy either by acting as an early alarm system via vibration sensing or by bringing the animal out of reach for nocturnal predators. We propose tractable hypotheses to test an adaptive function of suspended resting. Further studies will shed light on the sensory challenges that animals face during resting phases and should target the mechanisms and strategies by which such challenges are overcome.

**Supplementary Information:**

The online version contains supplementary material available at 10.1186/s12983-021-00410-3.

## Background

Most animals seek out safe sites during resting and sleeping phases, allowing them to lower their metabolic rates and be minimally vigilant [1]. Resting strategies and sites can reduce exposure to adverse abiotic (e.g., temperature) and biotic (e.g., predators) interactions [2], thus directly influencing fitness [3]. However, despite the ubiquity of this challenge, resting strategies and site-selection has thus far gained most attention in vertebrate species, where sleep/resting often takes up a substantial proportion of these animals’ lives [4]. Studies on invertebrates have either focused on related aspects such as background matching choice of moths [5, 6] or resting site selection of invertebrate vectors of diseases such as mosquito pupae or tsetse flies [7, 8].

For both diurnal and nocturnal animals, choosing a resting site with minimal exposure to both predators and environmental conditions is crucial [3]. For diurnal invertebrates that heavily rely on vision specialized for daylight conditions, we might expect that nocturnal resting strategies are particularly important due to the sensory disadvantage faced at night. Specifically, when visual perception of potential threats is limited, we expect adaptive strategies that either allow perception via different or additional sensory modalities and/or selection for specific resting sites with an overall lower risk of predator encounter.

Salticids are visual specialists with rich cognitive and visual abilities [9]. Their large forward-facing principal eyes are capable of depth perception [10], colour vision [11], a spatial resolution that surpasses that of many vertebrates and have recently been shown to even perform well under dim-light conditions [12]. Yet, these eyes are just one of four total pairs, with the remaining six, smaller eyes providing a near 300° field of view and being especially adapted for motion detection [13, 14].

Jumping spiders visually detect, locate, and identify prey and predators. Most jumping spiders do not commonly build webs or use silk to capture prey (for noteworthy exceptions see [9, 15]). Salticids do, however, use silk for several other functions, including draglines to stabilize accurate jumps [16, 17] and to facilitate chemical communication [18, 19]. These spiders also construct silk retreats used for resting during the night, which are also occupied during the day, for moulting, egg deposition, and even for mating [20]. Across the family, the precise structure and location of these retreats varies, from leaf-rolling, platforms, tubular shelters including multiple entrances to suspension nests [21, 22].

The use of densely-woven silk retreats (e.g., silk tubes with openings at each end), however, is not the only nocturnal resting behaviour that has been observed in jumping spiders: resting upside down from a silk line attached to the vegetation was anecdotally noted for four salticid species *Lyssomanes* sp. [23], *Menemerus bivittatus* [24], *Thiodina* sp [25] and *Mopsus mormon* [26], although only Carroll [25] explicitly described it as a nocturnal resting behaviour. Apart from these brief observations, no quantitative documentation or functional discussion has followed. This lack of discussion in the literature is surprising, as this behaviour differs markedly from the retreat-based resting commonly attributed to salticids. Indeed, this phenomenon implies the existence of intriguing behavioural diversity and flexibility within jumping spiders, offering a powerful system in which to explore functional questions related to nocturnal behaviours and sensory challenges during that time.

**Fig. 1:**
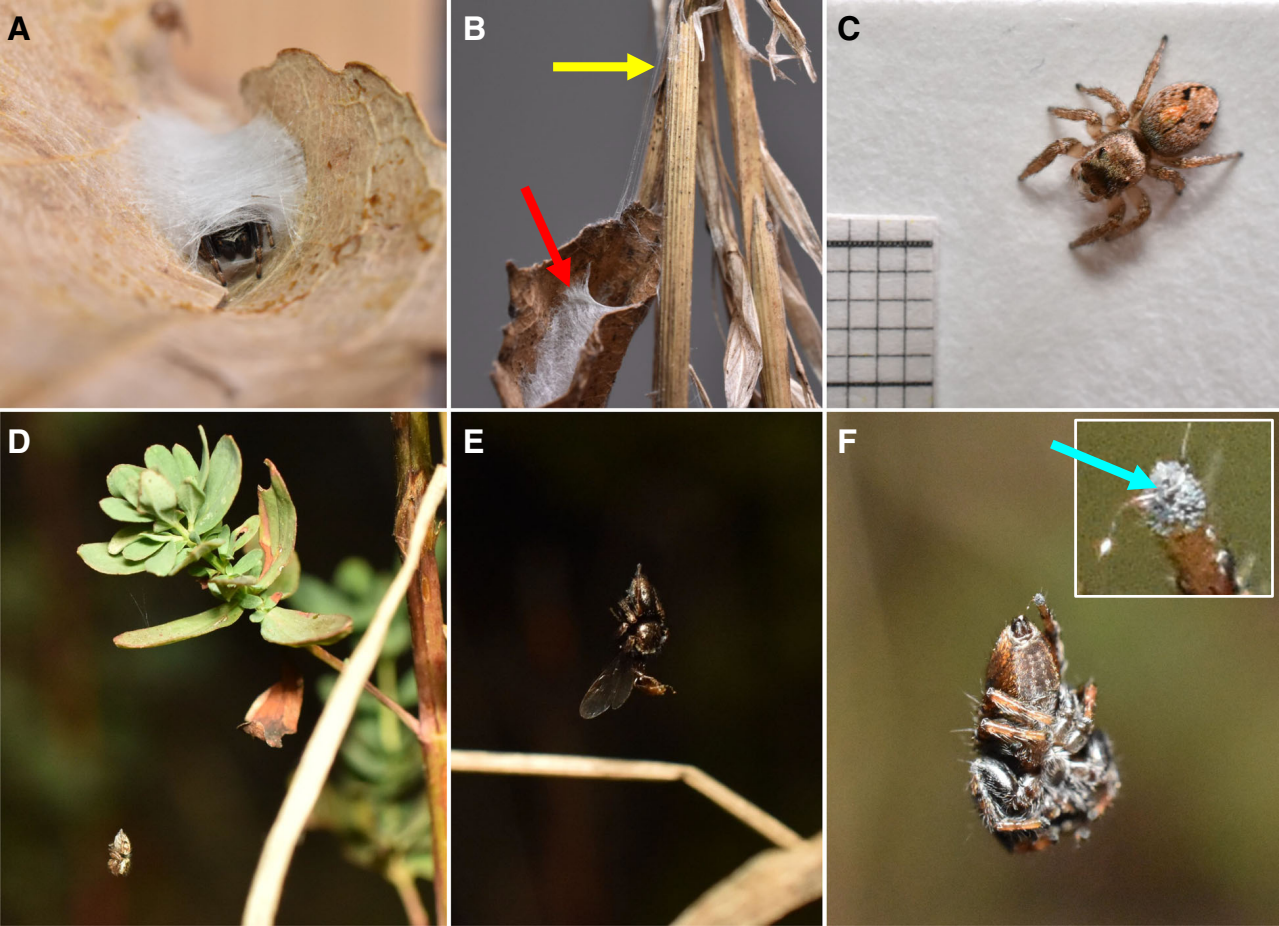
Photographs of *Evarcha arcuata* from the field and the laboratory. **a** Adult male *Evarcha arcuata* in typical silk retreat spun within a dead leaf (actively rolling the leaf together in the building process). **b** Silk tube retreat in a rolled-up leaf (red arrow) actively suspended and attached on vegetation (yellow arrow) by the spider. **c** Marked adult female with light coloration. **d** Subadult female hanging suspended from the vegetation. **e** Adult male with typical dark coloration hanging suspended while feeding on a prey item. **f** Suspended position with visible curve in silk line between spinnerets and tarsus. Close-up shows potential holding mechanism via two tarsal claws (blue arrow). **a***-***c** in captivity, **d***-***f** in the field

In September 2020, we observed this nocturnal suspension behaviour for the first time in the common European jumping spider *Evarcha arcuata* in the field in Trier, Germany (Fig. 1d and e). Despite having spent a lot of time in the field, we never observed hanging during the day, thus, this behaviour is probably only exhibited at night. Here, we provide the first data on this behaviour along with descriptions from both the field and the laboratory. We recorded hanging positions of all encountered spiders to test whether the behaviour differs between sexes and developmental stages. As a step toward clarifying the function of this spider’s nocturnal suspension behaviour, we preliminarily investigated the way suspended spiders responded to different disturbances and documented daily movement by the spiders. We provide a detailed description of this behaviour and discuss hypotheses about its function.

## Methods

### Data collection

**Fig. 2:**
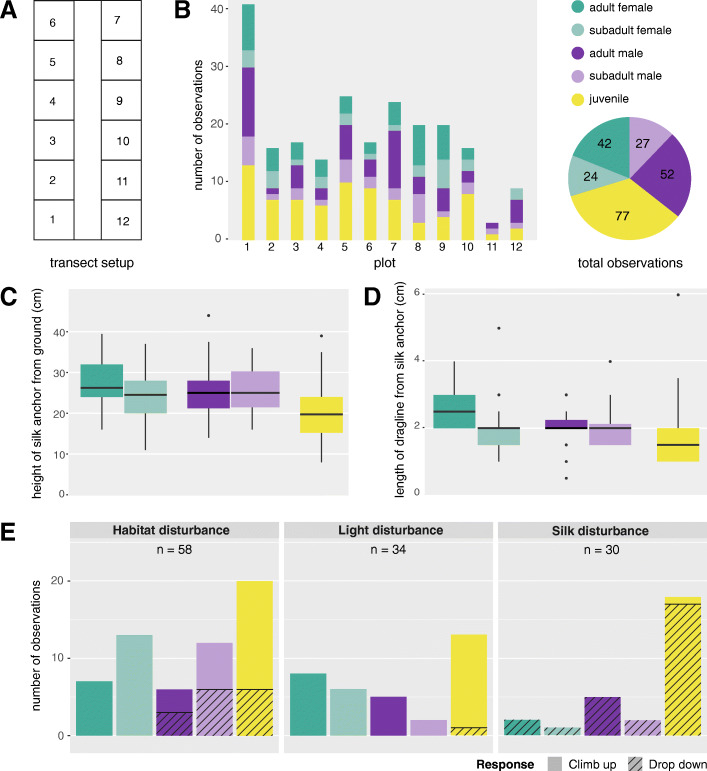
Descriptive data of the 9-day field study. **a** Field transect setup of 12 plots separated by a path in the middle. **b** Number, sex, and maturity of spiders found in each plot, pie chart showing sex and maturity distribution of all observations at field site (*N =* 222). **c** Boxplots of anchor positions during hanging for different sexes and developmental stages. **d** Boxplots of length of the hanging dragline for different sexes and developmental stages. Black lines represent the median, lower and upper bound of the boxes show 25th and 75th percentiles with whiskers representing ± 1.5 interquartile range. Black dots show outliers. **e** Number of observed responses (climbing up or dropping down) towards different disturbance stimuli for different sexes and developmental stages. Definitions of disturbances can be found in the methods

Observations and data collection were conducted on a patch of dry grassland in Trier, Germany (49°44’55.0"N, 6°40’39.9"E) between September 15 and 24, 2020. A total of 12 2 × 2 m square plots were marked (Fig. 2a, supplementary methods S1) and visited twice a day (diurnal and nocturnal monitoring) over the course of 9 days. Daytime visits focused on monitoring active spiders, and thus took place when weather conditions were favourable (warm and sunny), and spiders were likely to be active (usually between 9 am and 2 pm). Night monitoring focused on locating resting spiders and always took place between 9 pm and 11 pm (following sunset at around 8 pm). During each visit, two observers searched each plot for 5 min. Plots were randomly checked by a different observer to avoid bias.

### Sex, maturity, and height measurements

Sex (where possible) and maturity of each spider encountered was noted. Adult spiders could easily be sexed based on sexual colour dimorphism (dark males and light females, see Fig. 1e and c), subadult males and females were identified based on the presence of palpal swelling or the absence of an external epigyne in conjunction with colour and size of adults. All other individuals were categorized as juveniles without further classification of sex. When spiders were found in the hanging position, the height of the silk anchor and the height of the spider above ground were recorded. Based on these values, we subsequently calculated dragline length (length of the silk from the anchor to the spider). To assess the degree of site fidelity, we captured, labelled, and re-released spiders within the observation site, with each individual being marked with a unique colour label. Within-plot coordinates of all individuals encountered were recorded (for a full description of mark-recapture efforts, see supplementary methods S1).

### Disturbance response

To identify a potential function of the suspended position, we carefully documented behavioural responses towards different disturbances during our night monitoring. Responses to three forms of disturbances were documented after recording the previously listed data: (1) Habitat disturbance (HD): observer touched the surrounding vegetation leading to a shaking of the anchored vegetation, (2) light disturbance (LD): shining an electric torch at the spider, and (3) silk disturbance (SD): silk thread with the spider attached was gently touched with the right index finger by the observer. Except for two cases where disturbances did not trigger a direct response (based on 124 total observations), spiders responded in one of two ways: they either climbed up towards the anchor point or dropped down into the vegetation.

### Observations in captivity

We kept 27 spiders in captivity (adult female = 9, subadult female = 5, adult male = 5, subadult male = 5, juvenile = 3) to gather qualitative information on the sequence of the behaviour involved with suspended resting, as well as initiation time and duration. We recorded close-up videos of suspension initiation using a Nikon D7200 with an AF-S Micro Nikkor 40 mm lens placed around 5 cm in front of the plastic box. For 24-h time-lapse videos, we used an Apple iPad Air with an image interval of 2.5 s (Skyflow time-lapse app) placed in front of 12 plastic boxes each containing a spider (6 adult females, 6 adult males). Animals were kept singly in clear plastic boxes (6 × 6 × 16 cm, width × depth × height) enriched with vegetation from the natural habitat including dried grass (details, see supplementary methods S1: figure S4) and leaves and were fed with *Drosophila* (*ad libitum*). Moisture was provided by water-filled Eppendorf tubes stoppered with cotton wool. The light:dark regime in captivity was 14:10, mean temperature and relative humidity were 22 °C and 70 %.

### Statistical analysis

All analyses were carried out in R 3.6.2 [27]. First, we analysed the effect of sex and maturity of the spiders on the height of the anchor point and the length of the dragline, using a linear model. After observing the effects of the dependent variables, Tukey corrected post-hoc analyses were carried out using the package *emmeans* [28]. Subsequently, we tested the effect of disturbance on spiders’ responses using a generalized linear model with binomial error structure using the package *glmmTMB* [29]. We set the observed response as a binomial dependent variable (1 for climbing up the dragline, 0 for dropping) and set the disturbance type, sex and maturity as predictors. We used the package *car* [30] to observe the effects of the independent variables and followed up with post-hoc analyses using Bonferroni correction on those which had an effect. Model fit was confirmed using the package *DHARMa* [31]. All plots were generated using the package *ggplot2* [32]. The full analysis and all created plots are available as an R markdown script in the supplementary methods S2.

## Results

### Sex, maturity, and height measurements

We documented a total of 222 sightings of *E. arcuata*, 70 from diurnal and 152 from nocturnal monitoring at the study site. Distribution of males, females, and juveniles was about equal (Fig. 2b). Noteworthy is the high number of sightings in plot 1 and the low number of sightings in plot 11, which can most likely be ascribed to differences in vegetation density (dense in 1, sparse in 11). From the 152 nocturnal sightings, 146 are documented cases of resting by hanging by a silk thread, the remaining six spiders were encountered sitting on vegetation.

We recorded a mean height of the silk anchor of 23.4 cm (SD ± 7.1 cm) above the ground. Both males and females anchored at a significantly higher height than juveniles (linear model, Tukey corrected post-hoc. MalesVsJuveniles: est. = 4.41, SE = 1.36, t = 3.24, *P =* 0.005; FemalesVsJuveniles: est. = 5.85, SE = 1.4, t = 4.18, *P =* 0.0002), while there was no difference between males and females (est. = -1.44, SE = 1.51, t = -0.95, *P =* 0.72) (Fig. 2c). We found that dragline length for females was significantly longer than for juveniles (est. = 0.47, SE = 0.18, t = 2.55, *P =* 0.035), but not significantly different from dragline length for males (est. = 0.08, SE = 0.2, t = 0.38, *P =* 0.97). Male dragline length was not significantly different from dragline lengths of juvenile spiders (est. = 0.39, SE = 0.19, t = 2.07, *P =* 0.12) (Fig. 2d).

### Disturbance response

We collected information on behavioural responses to different disturbance stimuli in 122 spiders (Fig. 2e). For the LD condition, nearly all spiders climbed up (33/34, 97 %), while for the SD condition nearly all spiders dropped down (29/30, 96.6 %). Interestingly, we found that spiders behaved differently depending on sex for the HD condition. Females always climbed up (19/19, 100 %), while only half of the males did (11/21, 52.4 %). Because of this data separation (all females climbing up), our generalized linear model could not calculate statistically significant differences between the sexes (see supplementary methods S2). Moreover, roughly three quarters of juvenile spiders climbed up (13/18, 72.2 %), suggesting that this group may have been composed of half males and half females, a hypothesis supported by the roughly equal numbers of adult males and females in our observations (see Fig. 2b).

### Behavioural description

**Fig. 3:**
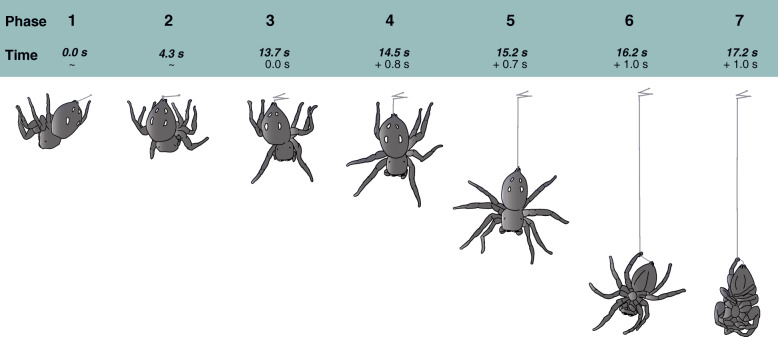
Illustration of the seven phases of suspended resting behaviour from initiation until final position

Based on videos taken in the laboratory and from field observations, we identified a sequence of seven phases of suspension-line building (Fig. 3, video S1): *Phase 1*: The first silk anchor is attached. *Phase 2*: A second anchor is attached in very close proximity to the first anchor, followed by around 2–4 (or more) anchors all of which are attached with a left-right (or vice versa) movement of the abdomen (time until all anchors are set can vary, typically taking approximately 6 to 13 s). *Phase 3*: With all anchors in place, the spider initiates dropping by orienting the anterior end of their cephalothorax downward, releasing the front pair of legs from the surface and holding them out-stretched, pointed downwards. *Phase 4*: The spider then releases the second pair of legs from the anchoring surface (in the laboratory this was the lid of the plastic container, in the field this was vegetation). *Phase 5*: All legs are released from the surface and the spider drops down on the dragline with all legs stretched out—descent during this phase is relatively continuous, likely controlled by muscles in the spinnerets that regulate the rate of silk production (cf. Wilson [33]). *Phase 6*: Dropping stops without legs touching the silk. Then, using one leg of the fourth pair of legs (Leg IV), the spider grabs the dragline just above the spinnerets, presumably with the two tarsal claws (Fig. 1f, cf. Kesel et al. [34]). The spider typically begins to rotate back and forth during this phase, about the axis of the silk thread. Initial rotations are ~ 180º and reduce (and reverse) with each partial rotation, suggesting a passive consequence of the line grabbing behaviour. *Phase 7*: All legs, except for the leg holding the silk line, are folded in. Initially during this phase, spiders often remain active, either cleaning themselves or even eating—we observed spiders suspended with prey items held in the spiders’ chelicerae several times in the field (prey included aphids, other spiders, and flies). In his observation, Carroll (1977) also noted that spiders hung by their thread in the evening “finishing their last meal of the day” [25]. It would be interesting to know when and how prey items found during these observations were caught by the spiders, however, we did not make any observations thereof. Once in this final phase, the silk between the spinnerets and the tarsal claws appears curved (Fig. 1f), suggesting that the forces required for suspension are applied by the claws, not the spinnerets. The process from first anchor to dropping was variable in time in our observations, typically taking around 15 s, with dropping to the final phase typically lasting 2-3 s (times in Fig. 3 averaged from 3 videos).

In the laboratory, the behaviour was initiated between 8 and 9 pm (sunset at around 7.30 pm during that time), which was in line with our finding that spiders were hanging suspended in the field by 8.30 pm. Four (1 adult female, 3 adult males) out of 12 spiders (6 adult females and 6 adult males) recorded in time-lapse in captivity remained suspended almost uninterrupted for an average of 5.6 h, until 2–4 am (*N =* 4, times in hours: 7.05; 4.54; 5.47; 5.49) (see video S2). Multiple instances of short hanging periods or interrupted hanging periods were observed for other spiders in the time-lapse video, which may have been due to disturbances (vibration, light) or the unnaturally smooth surface of the plastic boxes. In the field we also regularly observed that once disturbed, spiders that climbed up to the anchor position resumed the suspended resting position within minutes after a disturbance.

### Observations in captivity

Of the 27 spiders housed in captivity, 14 exhibited nocturnal resting by hanging on a silk thread at least once, typically using the smooth plastic box lid rather than the vegetation to anchor the silk thread. By the end of October, all spiders in captivity had built dense silk retreats attached to vegetation (Fig. 1b), which are used for resting, moulting and sheltering egg sacs. However, by the end of November we observed reversal switching, meaning that some spiders in captivity occasionally still suspended themselves from the lid of the plastic box despite having built a retreat which was present in the box.

Additionally, we were able to observe four out of six spiderlings that emerged from an egg sac (in April 2021) deposited by one of the females in captivity, taking on a suspended resting position on the very first night post- emergence (supplementary methods S1: figure S5).

## Discussion

Hanging suspended on a silk thread seems to be a common strategy for both sexes and all developmental stages (including newly emerged spiderlings) in *Evarcha arcuata*, yet these spiders are also capable of building typical salticid silk retreats, which we observed both in the field and in the laboratory (Fig. 1a, supplementary methods S1: figure S3). Thus, these spiders are clearly engaging in different strategies, even at the individual level. This behavioural plasticity in resting strategy is interesting, inviting questions such as why and how resting options are chosen and what factors inform this behavioural choice.

Perhaps the most noteworthy feature of this behaviour is that it appears to provide little, if any, protection from unfavourable abiotic environmental conditions—even less than if a spider were to simply remain stationary on a surface where it could receive thermal benefits or reduced exposure to air-movements due to boundary-layer effects. Thus, biotic factors seem likely to play a more important role than abiotic ones, particularly that hanging from an overhead line might offer some form of protection from predators. Building typical silk retreats and using them for resting offers thermal benefits as well as a physical boundary from potential predators.

Our first consideration is the necessary swap in sensory modality between diurnal and nocturnal sensing. Being highly visual animals, but with no active mechanism to keep light from entering their eyes, jumping spiders showed a strong reaction towards light by terminating the hanging behavior quickly after light disturbance. Equally, in the laboratory, initiation of the behavior was observed shortly after overhead lights were turned off. These observations suggest that, not surprisingly, at greater light levels, the preferred engagement with the environment is visual. At night, visual sensory information available to them is reduced or even unavailable. Consequently, the predominant sensory modality at night is likely vibration, the other main sensory modality used by spiders [35]. This is in line with findings that even courtship displays, usually thought of as predominantly visual, can switch with males performing tactile, vibratory courtship on the silk of female retreats that are located in low light conditions (e.g., under rocks) [20]. Vibrations can thus either be sensed directly via substrate-borne cues or silk-borne cues directly at the retreat, or, in the case of suspended resting, via a single silk thread held by the tarsal claws of the spider.

Spiders reacted differently towards shaking of the vegetation (habitat disturbance) versus silk disturbance. This may be intuitive—if there is a non-direct disturbance nearby, climb up and wait, but if there is a direct disturbance of the silk line, the only path to safety is to drop rather than to climb up. However, consideration of the sensory information available to the spider in these circumstances suggests that this differential response is non-trivial. Specifically, silk-transferred vibrational cues seem to be the most likely sensory modality involved in detecting both types of events, although we cannot completely rule out that spiders perceived additional visual cues posed by the observer’s hand via their lateral and posterior eyes. We also observed spiders hanging in the field even in windy conditions (> 1.5 m/s), clearly undisturbed by the wind-induced oscillations, suggesting that they can disregard wind-borne vibrations. Thus, these differential responses suggest that these spiders can discriminate between similar stimuli within this modality. The question of how spiders discriminate between silk-borne vibrational cues is particularly interesting because we also have evidence that the response to disturbance is sex-specific, as: 50 % of male spiders dropped in response to the habitat disturbance, while all females climbed up. Substrate-borne and silk-borne vibration-based signal differentiation is described among salticids, particularly in the context of sexual selection [36, 37] and is comparable to evidence of silk-based vibration discrimination in spider lineages that use silk in prey capture (e.g., Mortimer et al. [38]). We should note that the disturbance tests performed in our study were intended as preliminary.

Behaviourally, dropping to the ground in response to vegetation vibration seems rather costly given the disadvantage of reduced sight and the potentially increased probability of encountering predators. Dropping, however, is, a common anti-predator strategy in various non-salticid spiders [39, 40] as well as in other groups such as aphids, lepidopterans or ladybirds [41], underlining its potential anti-predator function. In order to establish an anti-predator function of suspended resting, a combination of the following would need to be demonstrated: increased survival by hanging overhead compared to fixed retreats and resting exposed on vegetation; increased use of overhead silk lines when the risk of predation is higher; a temporal overlap of overhead hanging with the peak activity of nocturnal predators.

Hanging suspended from the vegetation has been studied in parasitoid wasps, where cocoon suspension was shown to be an efficient strategy against foraging ants, however not against hyperparasitoids [42]. Hyperparasitoids track the silk on the vegetation and follow down the thread to the cocoon [42]. Based on our results, this type of behaviour would probably induce spiders to drop. However, we lack information on nocturnal parasitoid wasps at this field site. We did, however, repeatedly observe ants foraging at night at the field site, including attacks on crab spiders, thus, suspension against foraging ants is plausible. We also observed other potential nocturnal predators during our field observations, including various larger spider species, such as nursery web spiders (*Pisaura mirabilis*), crab spiders (*Xysticus* sp.) and yellow sac spiders (*Cheiracanthium punctorium*). Another study showed that caterpillars initiate suspending themselves on a silk thread in response to invertebrate predators while adjusting silk length to predator type [43]. Caterpillars can discriminate different predators based on substrate-borne vibrational cues, thus, a similar discrimination ability in jumping spiders is not far-fetched. A suspended position is likely to be an efficient anti-predator strategy, potentially acting as an early warning system or simply by bringing the spider out of reach for some predators. In his observation, Carroll (1977) further notes suspended resting during the night for two non-salticid spiders, the yellow crab spider (*Misumenops lepidus*) and the brown lynx spider (*Oxyopes scalaris*) both of which are also day-active [25]. This indeed suggests a more common strategy specially adapted for the night-time and potentially against nocturnal predators.

An alternative explanation is that suspended resting could increase dispersal distance, while reducing the temporal, energetic and cognitive costs of building, remembering, and returning to typical silk retreats. Considering that these spiders move several meters across highly structured and complex three-dimensional habitat, the ability to navigate back to an exact location (i.e., retreat), requires elaborate three-dimensional navigation, which is assumed to be cognitively demanding and costly. Consequently, spiders could maximise time investment in foraging and finding mates by dropping on a silk line at the end of the day. For that very reason, we initially hypothesised that, in search of mates, males move farther than females. Our preliminary data did not corroborate this hypothesis, but instead suggests that neither sex did show strict site fidelity (for a full description see supplementary methods S1). More data is needed, however, to test this preliminary finding.

The apparent plasticity in the nocturnal resting strategy of *Evarcha arcuata*, invites future investigations of resting strategies as well as resting site selection in adaptive contexts among invertebrates more broadly. We believe that this is a promising system to explore functional questions of how sensory information shapes behavioural choices. The functional link between predator-avoidance during phases in which main sensory paths are compromised is an interesting question particularly in diurnal invertebrates. Future studies in such systems are likely to gather novel insights into sensory information processing, resting site selection, and the fundamental role of resting.

Resting in the animal kingdom is associated with reduced activity (lower metabolic rates) [1], protection from unfavourable abiotic conditions as well as protection from predators [2]. Yet, most studies investigating resting so far have focused on vertebrate species. We have reason to believe that resting in invertebrate species follows similar concepts and rules, yet very little is known about the night-time activities of even common species such as *E. arcuata*. Gathering rigorous data on the exact sites that invertebrates choose for resting and what selective benefits these sites offer from an adaptive point of view will contribute valuable information for a more comprehensive understanding of resting in animals.

## Conclusions

Our work provides a detailed description of suspended resting in a common jumping spider. The described behaviour potentially is an effective anti-predator strategy either by acting as an early alarm system via vibration sensing or by bringing the animal out of reach for nocturnal predators. Considering that this behaviour has been anecdotally noted in at least four different species across four continents and given these species’ phylogenetic relationship, this strongly suggests that the behaviour has evolved independently multiple times across the salticids or is far more widespread than currently known. This also suggests that, if indeed driven by predator selection, potential predators are likely pervasive species with uniform nocturnal foraging modes (e.g., ants or parasitoids).

## Supplementary Information

**Additional file 1: Supplementary methods S1 and S2.** S1: Additional methods and supplementary figures S1 to S5; S2: R-Script of data analysis.

**Additional file 2: Dataset S1**. Data generated and analysed during the study.

**Additional file 3: Video S1**. Video of two hanging sequences.

**Additional file 4: Video S2.** Time-lapse video.

## Data Availability

The datasets generated and analysed during the current study as well as the complete R script are included in this published article and its supplementary information.
