# Supplementary material for: Hanging by a thread: unusual nocturnal resting behaviour in a jumping spider
Source: Front Zool. 2021 May 17;18:23. doi: 10.1186/s12983-021-00410-3 (PMC8127284; doi:10.1186/s12983-021-00410-3)
Supplement: Supplementary file 1 — Additional file 1: Supplementary methods S1 and S2. S1: Additional methods and supplementary figures S1 to S5; S2: R-Script of data analysis. [file 12983_2021_410_MOESM1_ESM.zip › Supplementary methods S1.pdf]

Supplementary information for

Hanging by a thread: unusual nocturnal resting behaviour in a jumping spider

Daniela C. Rößler[1], Massimo De Agrò[1], Elia Biundo[2], Paul S. Shamble[1]

[1]John Harvard Distinguished Science Fellows Program, Harvard University, Cambridge, MA 02138, USA

[2]Department of Biogeography, Trier University, 54295 Trier, Germany

Supplementary methods S1

Material and methods

Retreat site fidelity

It is generally assumed that salticids reuse retreats[1],[2]. Consequently, despite offering shelter, silk retreats in jumping spiders are likely associated with costs such as time and energy investment in structure building as well as the cognitive and energetic demand of choosing a suitable location or locating a previously built shelter. Thus, we initially hypothesized that the hanging behaviour may be linked to space use, as not having a fixed retreat to return to and being able to drop on a silk line at the end of the day, could increase overall dispersal distance. Consequently, we collected data on individual movement. For this purpose, a total of 35 individuals (11 adult females, 1 subadult female, 19 adult males, 2 subadult males and 2 juveniles) were captured and marked with unique colour codes (figure S1) using nontoxic acrylic paint (Talens art creation; Royal Talens). We documented recaptures during the 9-day study (recapture meaning re-

---

[1] Hoefler, C. D., and E. M. Jakob. 2006. Jumping spiders in space: movement patterns, nest site fidelity and the use of beacons. Animal Behaviour 71:109–116.

[2] Mooney, K. A., and J. R. Haloin. 2006. Nest site fidelity of *Paraphidippus aurantia* (Salticidae). The Journal of Arachnology 34:241–243.

identification without capturing the animal again). Twenty-three animals were released randomly across all plots on the first day of the study. Three days into the study an additional 12 individuals were released (figure S2). Points were measured using distance to plot centre and one of two reference points, providing accurate location information to approximately 10 cm scale.

Statistical analysis

See main manuscript for description on linear model. For the retreat site fidelity, we analysed the effect of sex and maturity on the distance travelled per day.

Result

During the nine days of our study, we re-identified 48.7 % of all marked animals at least once again. The maximum recapture of one individual was 5 times. On average, spiders moved 114 cm per day and moved a total distance of 228 ± 192 cm (mean ± SD). The minimum and maximum distance from the release point ranged from 21 to 737 cm. We found that sex (Linear model, Analysis of variance, $p = 0.3433$) had no effect on distance travelled per day from the release point.

Discussion

Our data on individuals' movements are incomplete as half of the marked individuals could have lost their marking, have been preyed upon or have dispersed far from the release points, out of the transects. Looking at the figure showing all recapture events and distances travelled (figure S2), we do suspect that in *E. arcuata* both sexes move extensively and do not have a fixed resting site that they return to. Consequently, overhead hanging as a potential "drop where you are"- strategy during their main activity period between May and September might be beneficial by maximizing time investment in mating and foraging. While we initially expected to see this behaviour to be more common in males, who roam for females, we found no such difference.

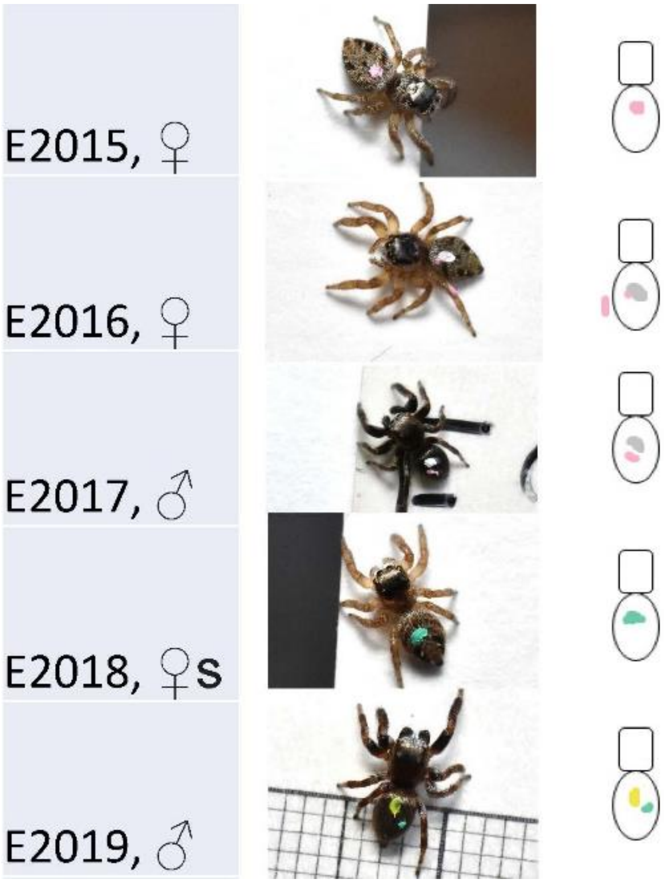

Figure S1. Examples of colour codes used in marking of spiders.

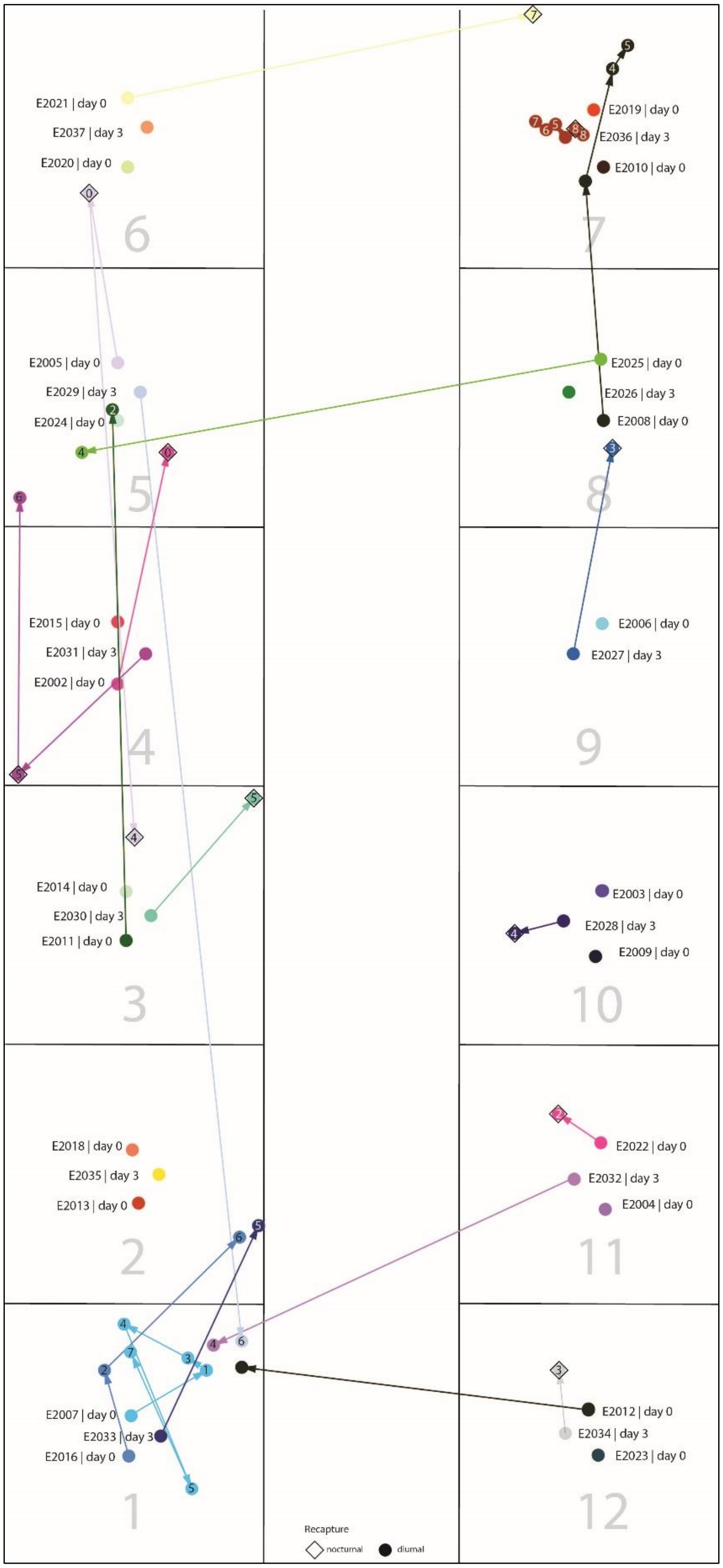

Figure S2. Transect of the field site showing the 12 4sqm plots with release points of marked spiders and subsequent recapture events (Circles refer to diurnal, squares to nocturnal recaptures; numbers show the day of the recapture event from 0 being the first day of the study).

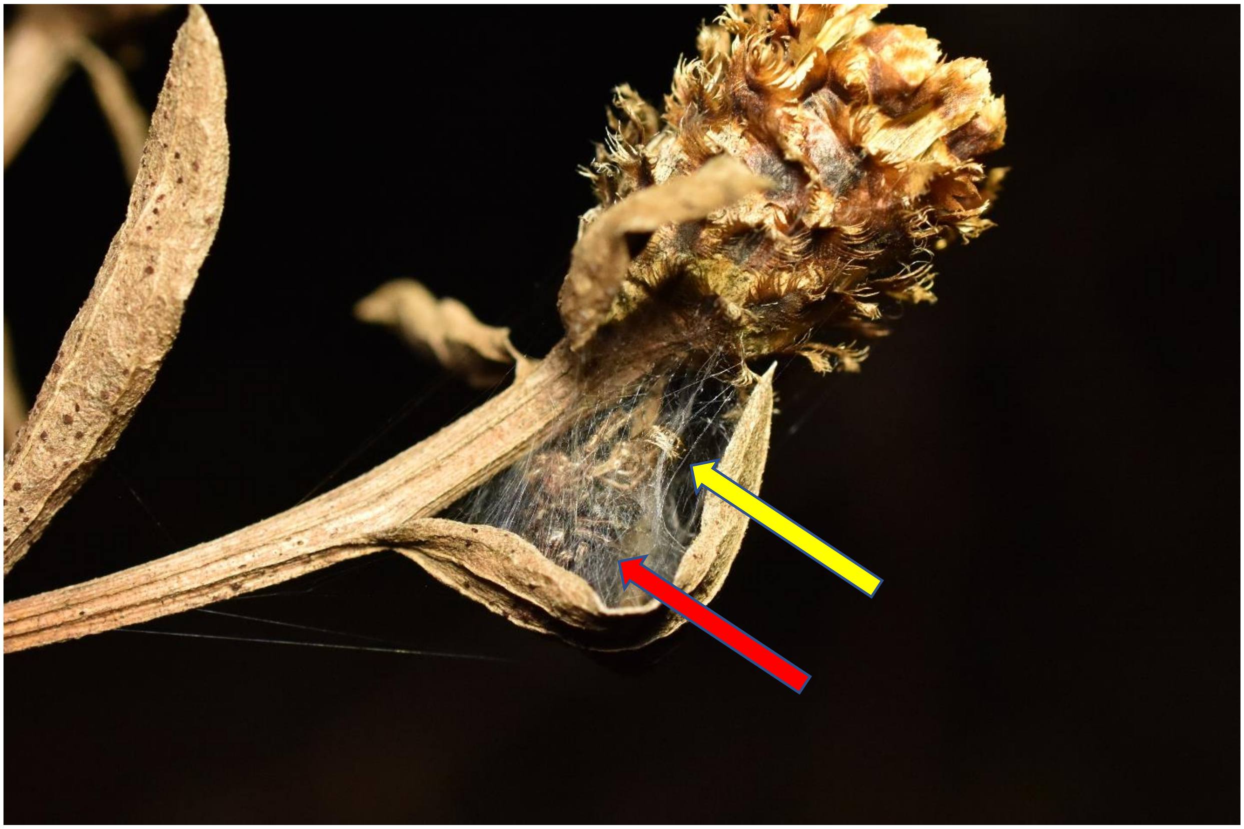

Figure S3. Moulting retreat of a male *Evarcha arcuata* in the field. Yellow arrow: moult of the spider, red arrow: male *Evarcha arcuata*.

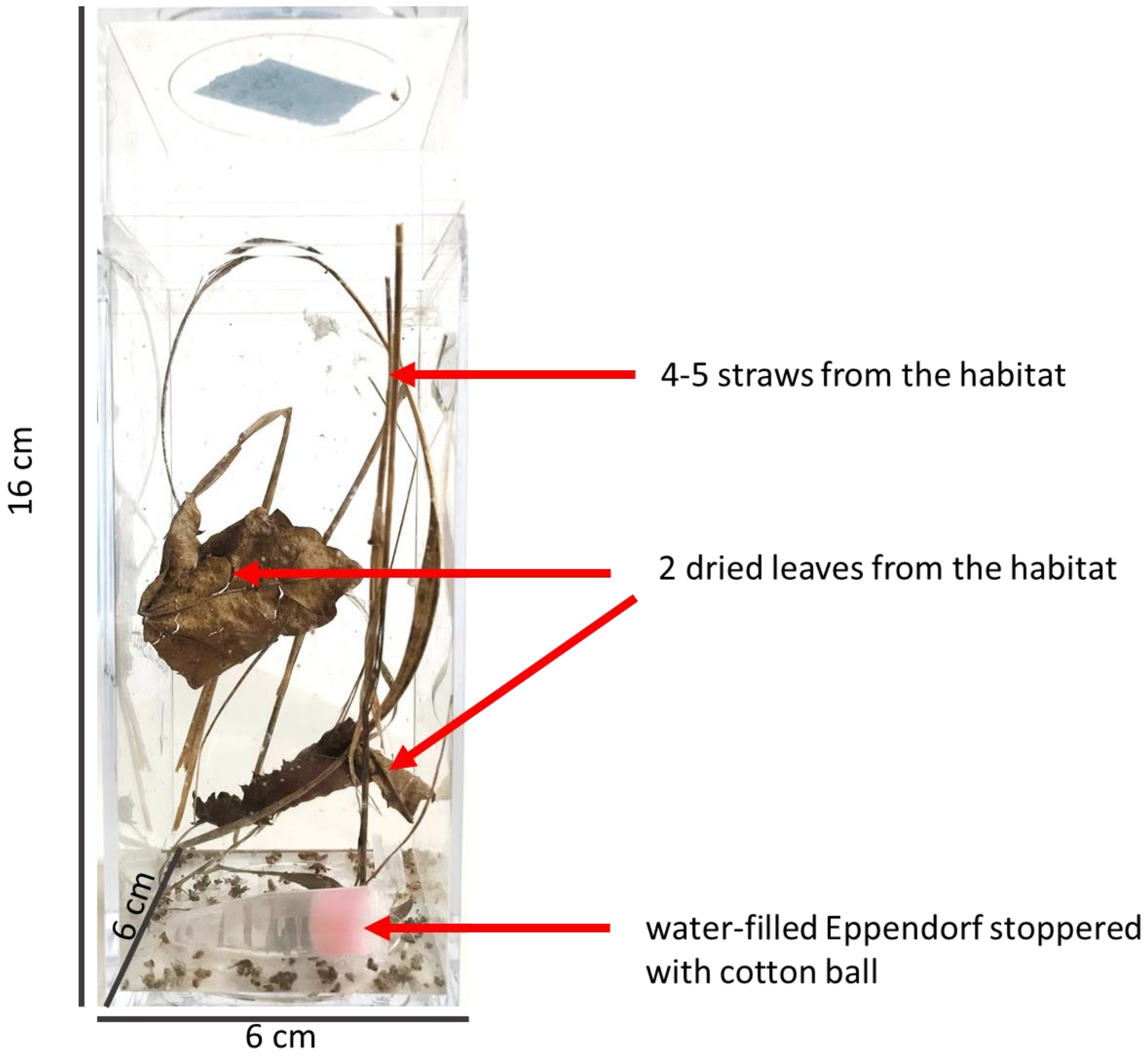

Figure S4. Plastic box used for spider housing including vegetation enrichment and water supply.

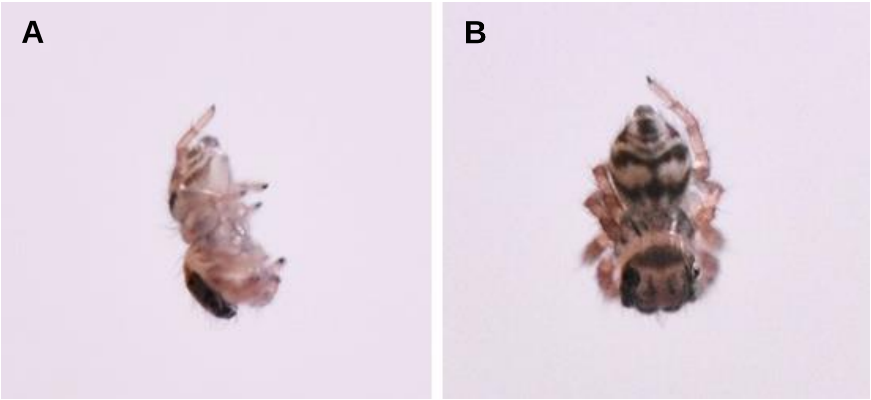

Figure S5. Spiderling (One day post-emergence from the eggsac, ~2mm in size) taking on a suspended resting position at night (*A*, side view; *B*, dorsal view).
