# Supplementary material for: Hanging by a thread: unusual nocturnal resting behaviour in a jumping spider
Source: Front Zool. 2021 May 17;18:23. doi: 10.1186/s12983-021-00410-3 (PMC8127284; doi:10.1186/s12983-021-00410-3)
Supplement: Supplementary file 1 — Additional file 1: Supplementary methods S1 and S2. S1: Additional methods and supplementary figures S1 to S5; S2: R-Script of data analysis. [file 12983_2021_410_MOESM1_ESM.zip › Supplementary methods S2.pdf]

Supplementary information for

Hanging by a thread: unusual nocturnal resting behaviour in a jumping spider

Daniela C. Rößler[1], Massimo De Agrò[1], Elia Biundo[2], Paul S. Shamble[1]

[1]John Harvard Distinguished Science Fellows Program, Harvard University, Cambridge, MA 02138, USA

[2]Department of Biogeography, Trier University, 54295 Trier, Germany

Supplementary methods S2

Supplementary methods S2 - Data Analysis

Daniela C. Rößler, Massimo De Agrò, Elia Biundo, Paul S. Shamble

03 December 2020

Abstract

This supplement provides the entire R script and output of the statistical analysis we performed and figures produced, in their original form. It is presented in the spirit of open and transparent science, but has not been carefully curated.

Preparation

Load packages

```
library(glmmTMB) #for mixed models
```

```
## Warning: package 'glmmTMB' was built under R version 3.6.3
```

```
library(DHARMa) #for goodness of fit test
```

```
## Warning: package 'DHARMa' was built under R version 3.6.3

## This is DHARMa 0.3.2.0. For overview type '?DHARMa'. For recent changes, type news(package = 'DHARMa
```

```
library(car) #for ANOVA and ANODA on mixed models
```

```
## Warning: package 'car' was built under R version 3.6.3

## Loading required package: carData

## Registered S3 methods overwritten by 'car':
##   method                          from
##   influence.merMod                lme4
##   cooks.distance.influence.merMod lme4
##   dfbeta.influence.merMod         lme4
##   dfbetas.influence.merMod        lme4
```

```
library(emmeans) #for post-hoc analysis
```

```
## Warning: package 'emmeans' was built under R version 3.6.3
```

```
library(ggplot2) #for graphs
```

```
## Warning: package 'ggplot2' was built under R version 3.6.3
```

```
library(fitdistrplus)
```

```
## Warning: package 'fitdistrplus' was built under R version 3.6.3

## Loading required package: MASS

## Loading required package: survival
```

```r
library(survival)
library(RColorBrewer)
```

Load dataset

```r
data <- read.csv('C:/Users/roess/Desktop/Publications/Evarcha/Supplements/ESM01-RawData.csv')
```

```r
head(data)
```

```
##        date        time spiderID marked obs_type  sex maturity sex_maturity
## 1 9/15/2020 11:00-12:00    E2002    yes  release male    adult   adult male
## 2 9/15/2020 22:00-23:00    E2002    yes    catch male    adult   adult male
## 3 9/15/2020 11:00-12:00    E2003    yes  release male    adult   adult male
## 4 9/15/2020 11:00-12:00    E2004    yes  release male    adult   adult male
## 5 9/15/2020 11:00-12:00    E2005    yes  release male    adult   adult male
## 6 9/15/2020 22:00-23:00    E2005    yes    catch male    adult   adult male
##   plot heightanchor heightspider heightdrop draglength disturbance response
## 1    4           NA           NA         NA         NA        <NA>       NA
## 2    5           NA           20         NA         NA        <NA>       NA
## 3   10           NA           NA         NA         NA        <NA>       NA
## 4   11           NA           NA         NA         NA        <NA>       NA
## 5    5           NA           NA         NA         NA        <NA>       NA
## 6    6           NA           15         NA         NA        <NA>       NA
##   releaseday startplot plotfound dayfound daypassed coordall_x coordall_y
## 1          0         4         4      0.0       0.0         86        521
## 2          0         4         5      0.5       0.5        125        342
## 3          0        10        10      0.0       0.0        460        681
## 4          0        11        11      0.0       0.0        463        927
## 5          0         5         5      0.0       0.0         86        273
## 6          0         5         6      0.5       0.5         62        142
##   coordplot_x coordplot_y distperday     dist comment
## 1          86         121         NA       NA
## 2         125         142   366.3987 183.1993
## 3         110          81         NA       NA
## 4         113         127         NA       NA
## 5          86          73         NA       NA
## 6          62         142   266.3607 266.3607
```

Analysis

Description of the hanging behavior

having observed *Evarcha* hanging during night rest, we collected data in nature regarding the height of the anchor point, the silk line length, as well as the individual characteristics (sex and age). We will in this section explore these variables

first of all, let's observe the distribution of sex and ages in the sample we observed

```r
#subset to only include capture events, and to exclude release events in counting observations
sub <- subset(data, data$obs_type == "catch")
table(sub$sex_maturity)
```

```
##
##    adult female      adult male        juvenile subadult female   subadult male
```

```
## 42 52 77 24 27
```

```
ggplot(sub, aes(x=sex,fill=maturity))+
       geom_bar()
```

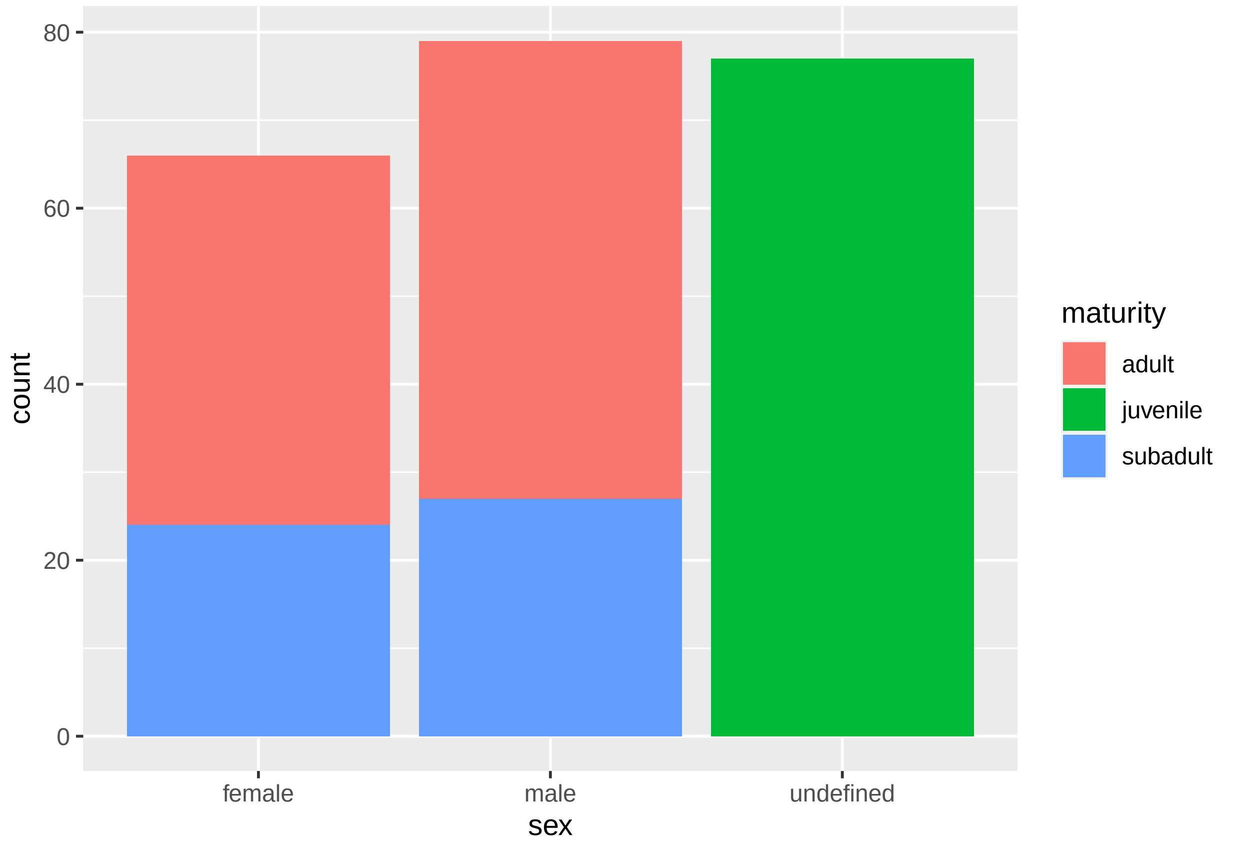

It seems like we found roughly the same number of males, females and juveniles. of the sexed spiders, we found more adult than subadults.

Let's now observe how these 5 groups affect behavior.

first, height of the anchor point

```
data$sex_maturity <- factor(data$sex_maturity,levels = c("adult male","subadult male", "adult female",

mh <- lm(heightanchor~sex*maturity, data)
anova(mh)
```

```
## Analysis of Variance Table
##
## Response: heightanchor
##               Df Sum Sq Mean Sq F value   Pr(>F)
## sex            2  904.4  452.22 10.0778 8.41e-05 ***
## maturity       1    7.8    7.80  0.1739   0.6773
## sex:maturity   1   50.5   50.46  1.1245   0.2909
## Residuals    133 5968.1   44.87
## ---
## Signif. codes:  0 '***' 0.001 '**' 0.01 '*' 0.05 '.' 0.1 ' ' 1
```

we found an effect of sex. We follow with a post-hoc. we keep maturity in as the post-hoc would produce

NAs otherwise, since no juvenile has a sex, and no sexed spider is juvenile

```
e <- emmeans(mh, ~sex*maturity)
e
```

```
##  sex       maturity emmean    SE  df lower.CL upper.CL
##  female    adult      26.0 1.498 133     23.0     28.9
##  male      adult      25.8 1.462 133     22.9     28.7
##  undefined adult    nonEst    NA  NA       NA       NA
##  female    juvenile nonEst    NA  NA       NA       NA
##  male      juvenile nonEst    NA  NA       NA       NA
##  undefined juvenile   20.5 0.872 133     18.8     22.2
##  female    subadult   23.8 1.462 133     20.9     26.7
##  male      subadult   26.9 1.625 133     23.6     30.1
##  undefined subadult nonEst    NA  NA       NA       NA
##
## Confidence level used: 0.95
```

```
contrast(e, list(FemalevsMale=c(0.5,-0.5,0,0,0,0,0.5,-0.5,0),
                 FemalevsUndefined=c(0.5,0,0,0,0,-1,0.5,0,0),
                 MalevsUndefined=c(0,0.5,0,0,0,-1,0,0.5,0)), adjust='tukey')
```

```
##  contrast          estimate   SE  df t.ratio p.value
##  FemalevsMale         -1.44 1.51 133 -0.951  0.7168
##  FemalevsUndefined     4.41 1.36 133  3.237  0.0046
##  MalevsUndefined       5.85 1.40 133  4.183  0.0002
##
## P value adjustment: sidak method for 3 tests
```

both males and females have a higher resting position than unsexed spiders. In other words, sexed spiders rest at higher altitude than juveniles.

now to plot

```
ggplot(data, aes(x=sex, heightanchor, fill=maturity))+
  geom_boxplot()+
  ylim(0,50)
```

```
## Warning: Removed 119 rows containing non-finite values (stat_boxplot).
```

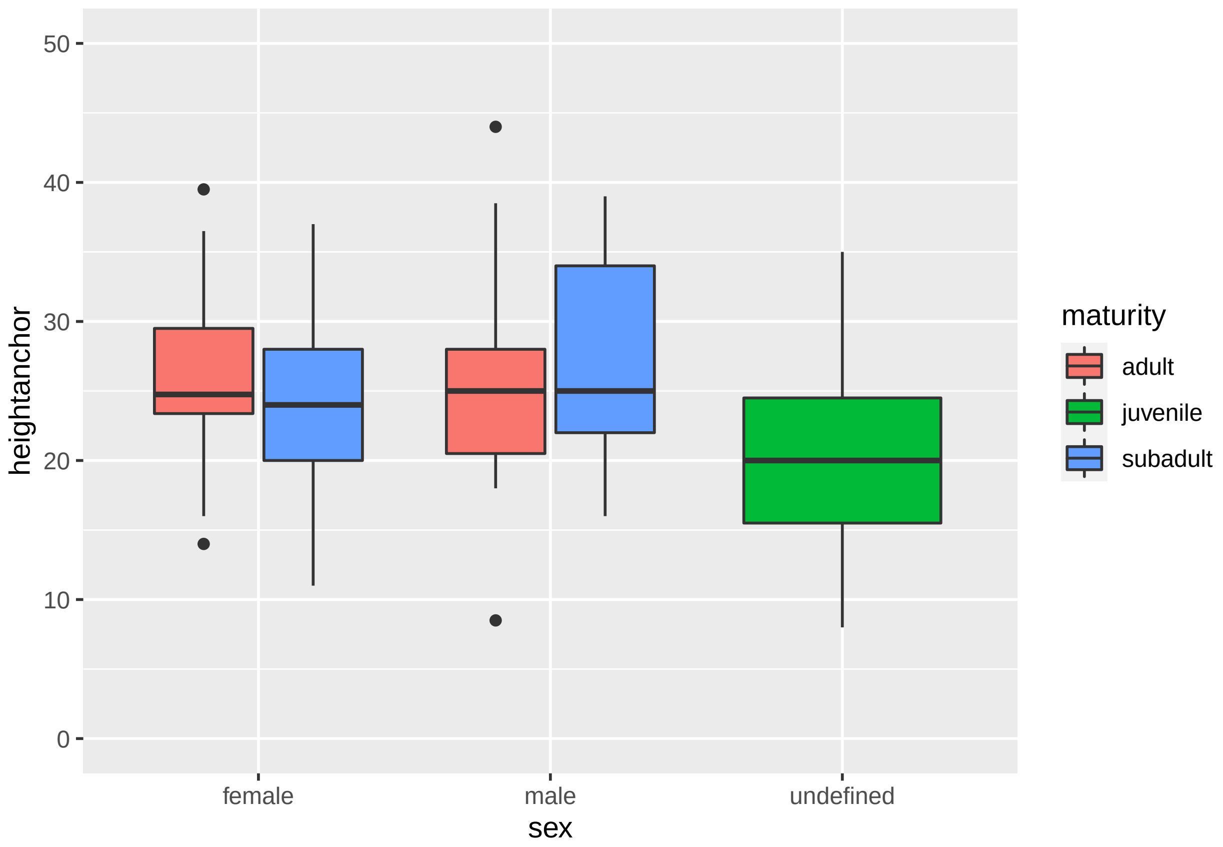

we observe no significant difference between sexes and ages for height of the anchor point

now, the dragline length

```
ml <- lm(draglength~sex*maturity, data)
anova(ml)
```

```
## Analysis of Variance Table
##
## Response: draglength
##                Df  Sum Sq Mean Sq F value  Pr(>F)
## sex             2   6.110 3.05482  3.7794 0.02533 *
## maturity        1   1.102 1.10157  1.3629 0.24513
## sex:maturity    1   2.732 2.73238  3.3805 0.06820 .
## Residuals     133 107.500 0.80827
## ---
## Signif. codes:  0 '***' 0.001 '**' 0.01 '*' 0.05 '.' 0.1 ' ' 1
```

we again found an effect of sex. post-hoc follows

```
e <- emmeans(ml, ~sex*maturity)
e
```

```
##  sex       maturity emmean    SE  df lower.CL upper.CL
##  female    adult      2.48 0.201 133     2.08     2.87
##  male      adult      2.02 0.196 133     1.64     2.41
##  undefined adult    nonEst    NA  NA       NA       NA
##  female    juvenile nonEst    NA  NA       NA       NA
##  male      juvenile nonEst    NA  NA       NA       NA
```

```
##  undefined juvenile   1.71 0.117 133     1.48     1.94
##  female    subadult   1.88 0.196 133     1.49     2.27
##  male      subadult   2.18 0.218 133     1.75     2.61
##  undefined subadult nonEst    NA   NA       NA       NA
##
## Confidence level used: 0.95
```

```
contrast(e, list(FemalevsMale=c(0.5,-0.5,0,0,0,0,0.5,-0.5,0),
                 FemalevsUndefined=c(0.5,0,0,0,0,-1,0.5,0,0),
                 MalevsUndefined=c(0,0.5,0,0,0,-1,0,0.5,0)), adjust='tukey')
```

```
##  contrast          estimate    SE  df t.ratio p.value
##  FemalevsMale        0.0778 0.203 133 0.383   0.9736
##  FemalevsUndefined   0.4661 0.183 133 2.549   0.0353
##  MalevsUndefined     0.3883 0.188 133 2.069   0.1165
##
## P value adjustment: sidak method for 3 tests
```

here, only females have a dragline longer than unsexed spiders. Multiple testing correction remove the significance in the male vs unsexed contrast.

```
ggplot(data, aes(x=sex_maturity, draglength, fill=sex))+
  geom_boxplot()+
  geom_jitter()+
  ylim(0,7.5)
```

```
## Warning: Removed 119 rows containing non-finite values (stat_boxplot).
```

```
## Warning: Removed 119 rows containing missing values (geom_point).
```

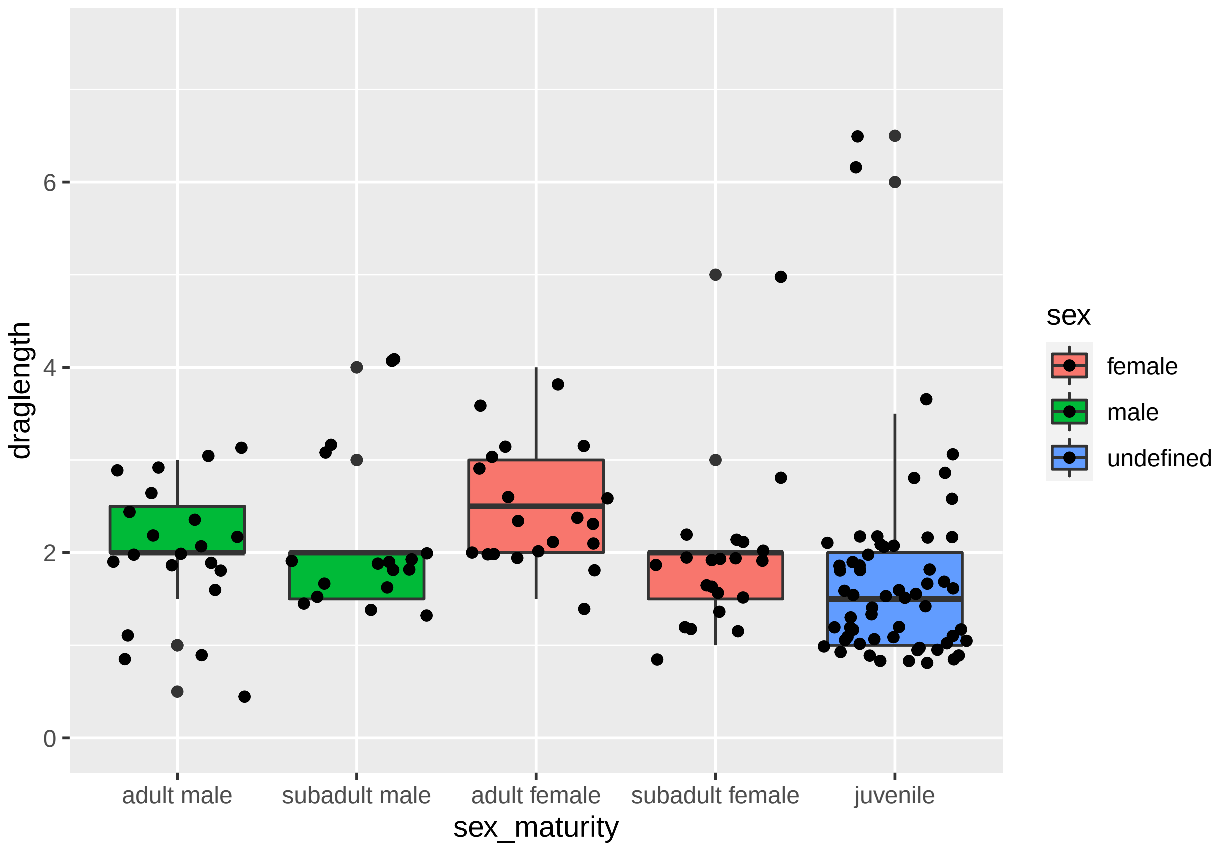

Reaction to external stimuli

We tested the reaction of Evarcha to different disturbances while hanging from the dragline. We caused 3 types of disturbances to the animals: we either moved branches near the spider (HD), touched the silk (SD) or shined a light on the animal (LD). We subjected each individual to only one of these conditions. We then recorded the response of the animal as a binomial, where the animal either climbed up the silk (1) or dropped down (0). no response was recoderd as NA

not that only two spiders didn't react to the disturbance.

```
md <- glm(response ~ disturbance * sex * maturity, data=data, family = binomial)
simres <- simulateResiduals(md)
plot(simres)
```

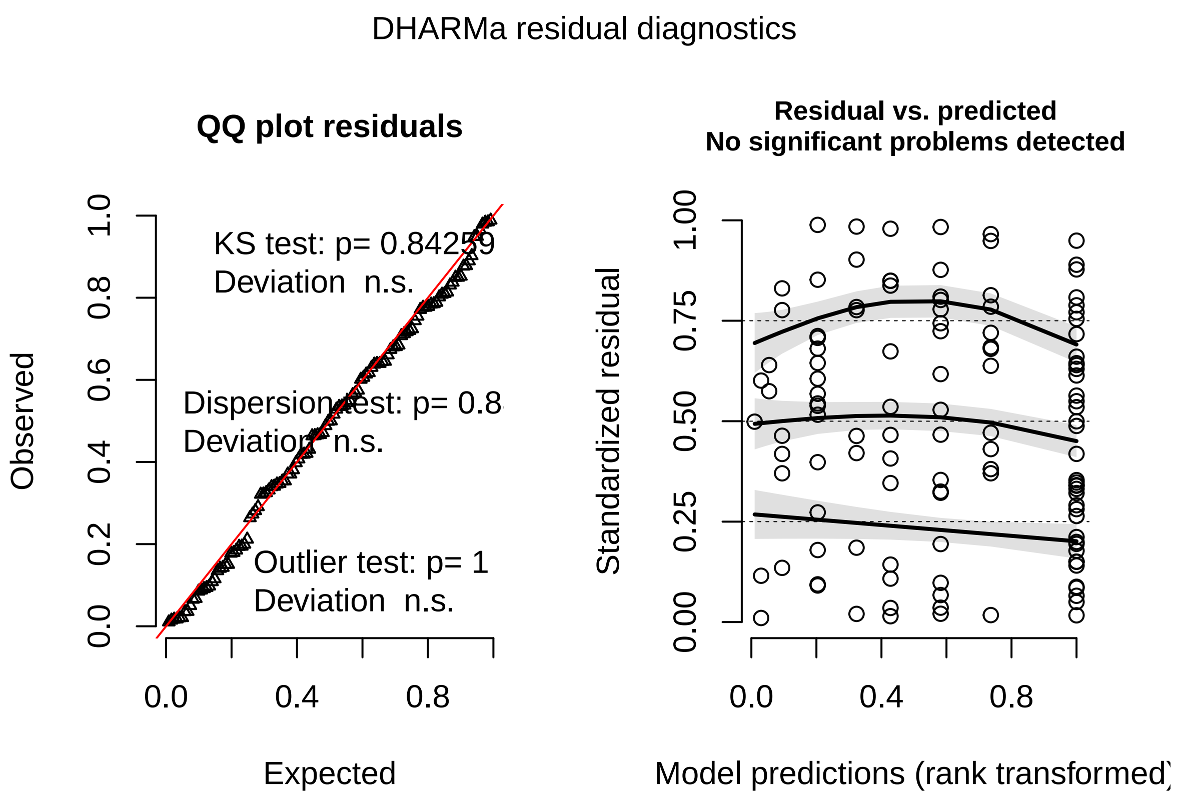

the distribution is correct

```
Anova(md)
```

```
## Warning: glm.fit: fitted probabilities numerically 0 or 1 occurred

## Warning: glm.fit: fitted probabilities numerically 0 or 1 occurred

## Analysis of Deviance Table (Type II tests)
##
## Response: response
##                            LR Chisq Df Pr(>Chisq)
## disturbance                  65.002  2  7.672e-15 ***
## sex                          15.916  1  6.621e-05 ***
## maturity                      0.029  1     0.8639
## disturbance:sex               0.000  2     1.0000
## disturbance:maturity          0.000  2     1.0000
## sex:maturity                  0.000  1     1.0000
## disturbance:sex:maturity      0.000  2     1.0000
## ---
## Signif. codes:  0 '***' 0.001 '**' 0.01 '*' 0.05 '.' 0.1 ' ' 1
```

there seems to be a difference between the different disturbances, and an effect of sex. Post-hoc

```
e <- emmeans(md, ~disturbance*sex*maturity, type='response')
e
```

```
##  disturbance sex      maturity     prob          SE  df asymp.LCL asymp.UCL
##  HD          female   adult    1.000000 0.00001293 Inf 0.0000000  1.000000
```

```
##  LD          female    adult    1.000000 0.00001140 Inf 0.0000000  1.000000
##  SD          female    adult    0.000000 0.00003421 Inf 0.0000000  1.000000
##  HD          male      adult    0.500000 0.17677670 Inf 0.2000625  0.799937
##  LD          male      adult    1.000000 0.00001530 Inf 0.0000000  1.000000
##  SD          male      adult    0.000000 0.00001397 Inf 0.0000000  1.000000
##  HD          undefined adult      nonEst         NA  NA        NA        NA
##  LD          undefined adult      nonEst         NA  NA        NA        NA
##  SD          undefined adult      nonEst         NA  NA        NA        NA
##  HD          female    juvenile   nonEst         NA  NA        NA        NA
##  LD          female    juvenile   nonEst         NA  NA        NA        NA
##  SD          female    juvenile   nonEst         NA  NA        NA        NA
##  HD          male      juvenile   nonEst         NA  NA        NA        NA
##  LD          male      juvenile   nonEst         NA  NA        NA        NA
##  SD          male      juvenile   nonEst         NA  NA        NA        NA
##  HD          undefined juvenile 0.722222 0.10557180 Inf 0.4810366  0.879416
##  LD          undefined juvenile 0.923077 0.07390530 Inf 0.6094287  0.989280
##  SD          undefined juvenile 0.062500 0.06051536 Inf 0.0087294  0.335412
##  HD          female    subadult 1.000000 0.00000988 Inf 0.0000000  1.000000
##  LD          female    subadult 1.000000 0.00001397 Inf 0.0000000  1.000000
##  SD          female    subadult 0.000000 0.00002419 Inf 0.0000000  1.000000
##  HD          male      subadult 0.538462 0.13826416 Inf 0.2816539  0.776359
##  LD          male      subadult 1.000000 0.00003421 Inf 0.0000000  1.000000
##  SD          male      subadult 0.000000 0.00001975 Inf 0.0000000  1.000000
##  HD          undefined subadult   nonEst         NA  NA        NA        NA
##  LD          undefined subadult   nonEst         NA  NA        NA        NA
##  SD          undefined subadult   nonEst         NA  NA        NA        NA
##
## Confidence level used: 0.95
## Intervals are back-transformed from the logit scale
```

by looking at the data, it is clear that all spiders but one (juvenile, by looking at percentage) climbed back up when disturbed by a light, and all spiders but one (juvenile again) dropped down when the silk was touched. It is clear that we will not see differences between sex and ages in these conditions, so we will only concentrate in the environment disturbance one

```
HD <- e[c(1,4,16,19,22)]
HD
```

```
##  disturbance sex       maturity     prob         SE  df asymp.LCL asymp.UCL
##  HD          female    adult    1.000000 0.00001293 Inf  0.000000  1.000000
##  HD          male      adult    0.500000 0.17677670 Inf  0.200063  0.799937
##  HD          undefined juvenile 0.722222 0.10557180 Inf  0.481037  0.879416
##  HD          female    subadult 1.000000 0.00000988 Inf  0.000000  1.000000
##  HD          male      subadult 0.538462 0.13826416 Inf  0.281654  0.776359
##
## Confidence level used: 0.95
## Intervals are back-transformed from the logit scale
```

```
contrast(HD, list(FemalevsMale=c(0.5,-0.5,0,0.5,-0.5),
                  FemalevsUndefined=c(0.5,0,-1,0.5,0),
                  MalevsUndefined=c(0,0.5,-1,0,0.5)),adjust='bonferroni')
```

```
##  contrast          odds.ratio       SE  df z.ratio p.value
##  FemalevsMale        2.91e+08 7.44e+11 Inf   0.008  1.0000
##  FemalevsUndefined   1.21e+08 3.09e+11 Inf   0.007  1.0000
##  MalevsUndefined     0.00e+00 0.00e+00 Inf  -1.269  0.6135
```

```
##
## P value adjustment: bonferroni method for 3 tests
## Tests are performed on the log odds ratio scale
```

regardless, we observe no significant difference between sexes and ages. It is unclear why, since by looking at raw data it seems obvious that females and males differ from one another: females always climb up, while males change strategy half the times. Probably the complete separation of data makes it impossible to test. indeed the confidence interval for the probability of 1 of females goes between 0 and 1, impossible to test significance in this scenario.

will plot for clarity

```
toplot <- as.data.frame(e)
ggplot(toplot, aes(sex, prob, color= maturity))+
  geom_point(cex = 3, position=position_dodge(.5))+
  geom_errorbar(aes(ymin=prob-SE,ymax=prob+SE), cex = 1, , position=position_dodge(.5))+
  facet_wrap(~disturbance)
```

```
## Warning: Removed 12 rows containing missing values (geom_point).
```

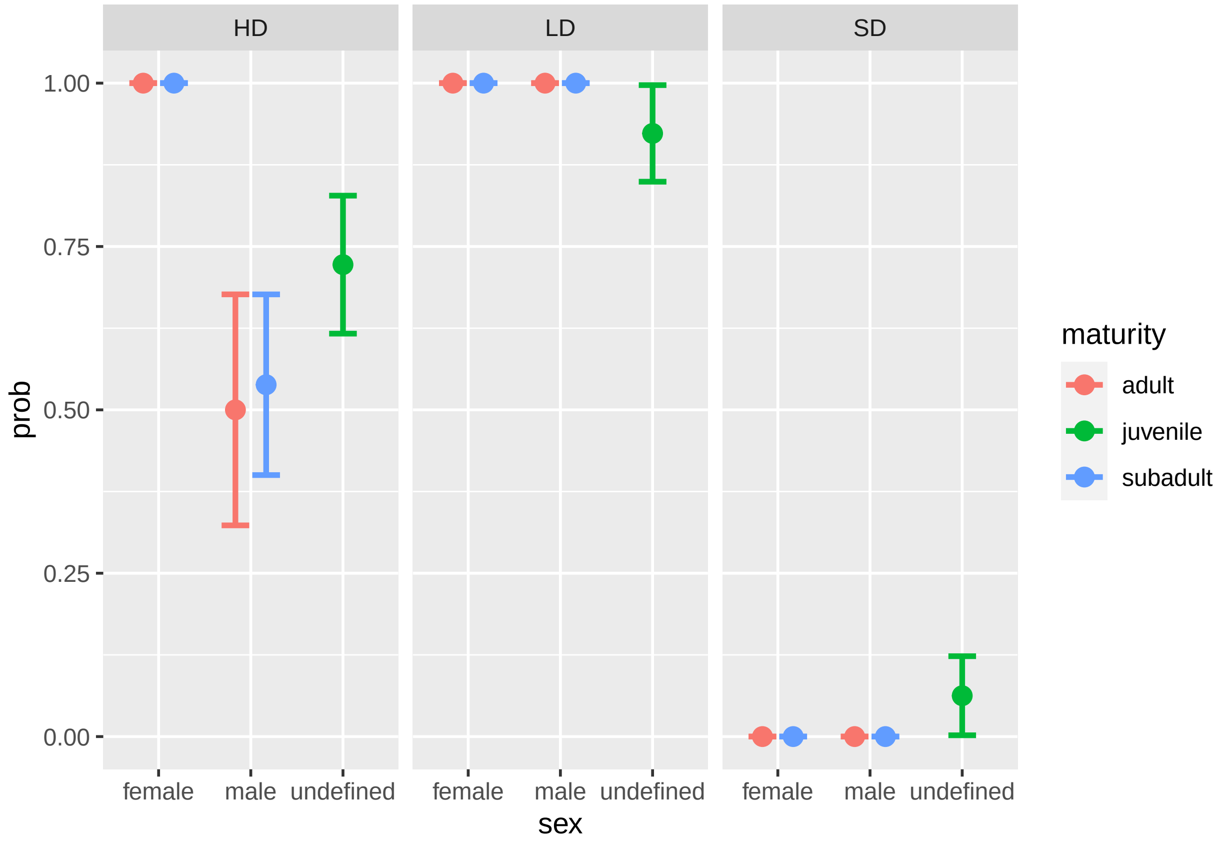

Site fidelity

lastly, we released marked Evarcha in the wild, and then proceeded to search in the same area to see if we were able to find them again. The underlying hypothesis for this test was that a hanging rest strategy would be consistent with a wandering lifestyle, while a classical cocoon would constitute a stable retreat to which the spider would return every day.

what is the average distance covered in one day by the spiders we retrieved? or in other words, how far did

we find them adjusted for day passed. Data is in centimeters.

```
md <- lm(distperday ~ sex * maturity, data = data)
anova(md)
```

```
## Analysis of Variance Table
##
## Response: distperday
##           Df Sum Sq Mean Sq F value Pr(>F)
## sex        2  32397 16198.7  1.1109 0.3433
## maturity   1   7660  7659.7  0.5253 0.4746
## Residuals 28 408284 14581.6
```

there does not seem to be a difference among sexes and ages, so will just look at the distribution overall.

```
hist(data$distperday, breaks = 5)
```

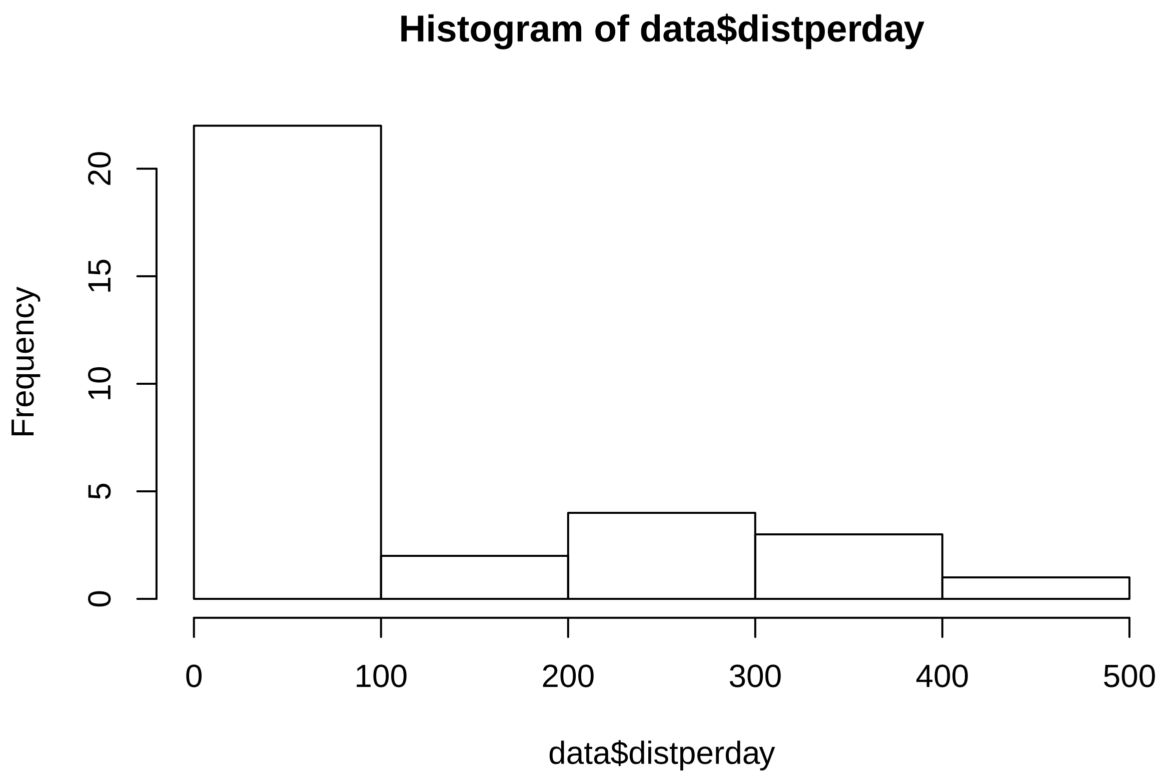

most of the recaptured animals moved less than 1 meter per day. However, it is possible to see a second peak around 3 meters per day.

now, will plot the raw positions of each animal

```
recaptured <- subset(data,data$marked=="yes")
ggplot(recaptured, aes(x=coordall_x,y=coordall_y, color=spiderID))+
        geom_point()+
        geom_line()+
        scale_y_reverse()
```

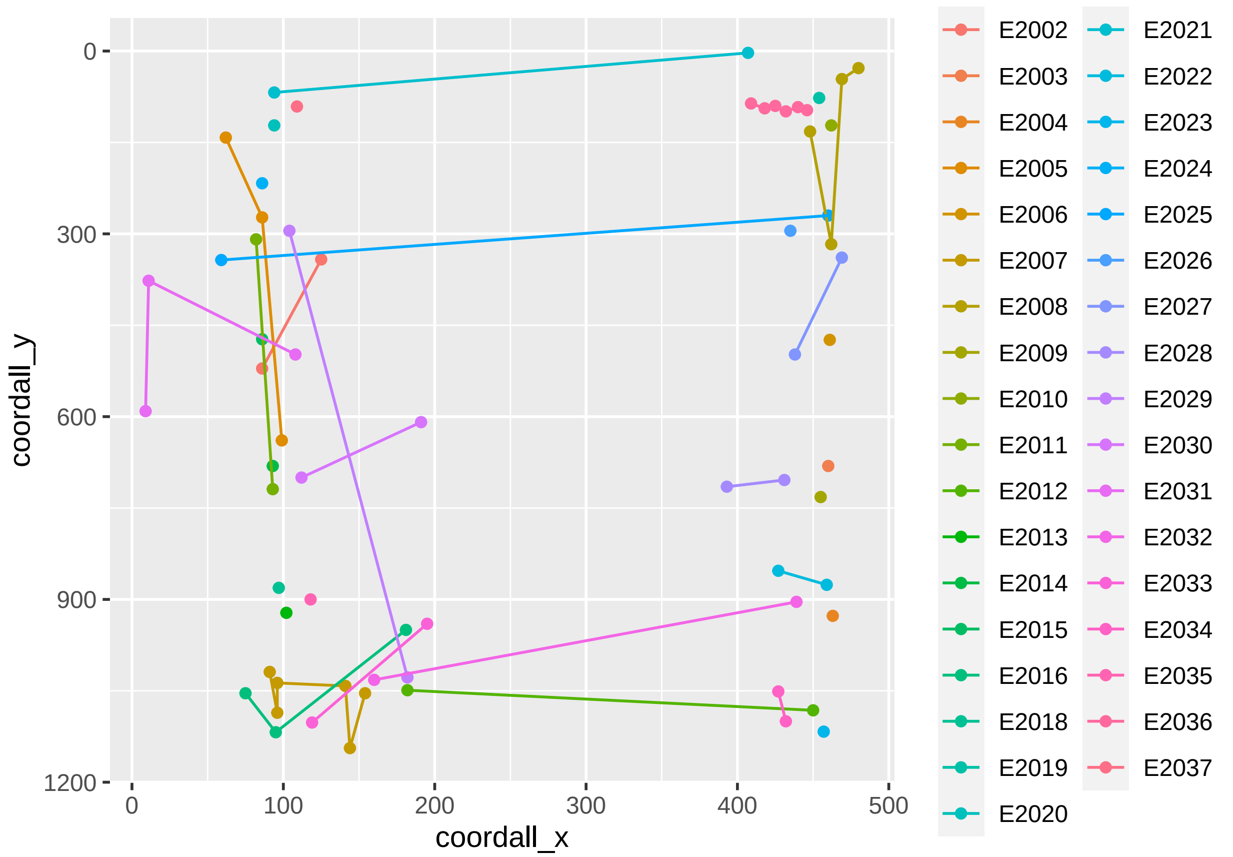

coordall_y
0
300
600
900
1200
coordall_x
0
100
200
300
400
500
E2002
E2003
E2004
E2005
E2006
E2007
E2008
E2009
E2010
E2011
E2012
E2013
E2014
E2015
E2016
E2018
E2019
E2020
E2021
E2022
E2023
E2024
E2025
E2026
E2027
E2028
E2029
E2030
E2031
E2032
E2033
E2034
E2035
E2036
E2037
